# Upper airway dynamic imaging during tidal breathing in awake and asleep subjects with obstructive sleep apnea and healthy controls

**DOI:** 10.14814/phy2.13711

**Published:** 2018-05-20

**Authors:** Chantal Darquenne, Ann R. Elliott, Bastien Sibille, Erik T. Smales, Pamela N. DeYoung, Rebecca J. Theilmann, Atul Malhotra

**Affiliations:** ^1^ Division of Physiology University of California San Diego California; ^2^ Division of Pulmonary Critical Care and Sleep Medicine University of California San Diego California; ^3^ Department of Radiology University of California San Diego California

**Keywords:** EEG, magnetic resonance imaging, natural sleep, obstructive sleep apnea

## Abstract

We used magnetic resonance imaging (MRI) to quantify change in upper airway dimension during tidal breathing in subjects with obstructive sleep apnea (OSA, *N* = 7) and BMI‐matched healthy controls (*N* = 7) during both wakefulness and natural sleep. Dynamic MR images of the upper airway were obtained on a 1.5 T MR scanner in contiguous 7.5 mm‐thick axial slices from the hard palate to the epiglottis along with synchronous MRI‐compatible electroencephalogram and nasal/oral flow measurements. The physiologic data were retrospectively scored to identify sleep state, and synchronized with dynamic MR images. For each image, the upper airway was characterized by its area, and linear dimensions (lateral and anterior–posterior). The dynamic behavior of the upper airway was assessed by the maximum change in these parameters over the tidal breath. Mean upper airway caliber was obtained by averaging data over the tidal breath. There was no major difference in the upper airway structure between OSA and controls except for a narrower airway at the low‐retropalatal/high‐retroglossal level in OSA than in controls. Changes in upper airway size over the tidal breath ((maximum − minimum)/mean) were significantly larger in the OSA than in the control group in the low retropalatal/high retroglossal region during both wakefulness and sleep. In the four OSA subjects who experienced obstructive apneas during MR imaging, the site of airway collapse during sleep corresponded to the region of the upper airway where changes in caliber during awake tidal breathing were the greatest. These observations suggest a potential role for dynamic OSA imaging during wakefulness.

## Introduction

Obstructive sleep apnea (OSA), a common sleep disorder with a prevalence of ~10% in the US population (~6% in women and ~13% in men) is characterized by intermittent partial or complete pharyngeal airway closure (Peppard et al. 2013). The causes and mechanisms of airway collapse are multifactorial and include upper airway anatomy, upper airway dilator muscle activity and reflex responsiveness, propensity for arousal from sleep, ventilatory control stability, and lung volume (Eckert and Malhotra 2008). Yet, based on current evidence, abnormal upper airway structure is likely the central component in the occurrence of OSA (Malhotra and White 2002; Yang and Woodson 2003).

The upper airway is a dynamic structure that changes throughout the respiratory cycle. These changes are affected by a number of conditions such as obesity, periodic breathing, wake and sleep state, and anatomical properties/abnormalities. Various imaging techniques, including fluoroscopy, cine computed tomography (CT), and magnetic resonance imaging (MRI) have been used in recent years to detect structural changes in the upper airway (Suratt et al. 1983; Shellock et al. 1992; Schwab et al. 1993a,b; Ciscar et al. 2001; Ikeda et al. 2001; Barkdull et al. 2008; Chuang et al. 2009; Wagshul et al. 2013; Kavcic et al. 2015). Among these, MRI provides the added advantage of monitoring dynamic changes by collecting multiple single‐shot real‐time images of the upper airway dynamics without concerns regarding ionizing radiation present in other imaging modalities such as CT.

Although previous studies have combined dynamic imaging of the upper airway with simultaneous quantitative monitoring of flow (Schwab et al. 1993a,b) or have combined imaging with electroencephalogram (EEG) recording to assess sleep stages (Kavcic et al. 2015), no study to date has combined dynamic imaging with both quantitative flow measurements and EEG sleep assessment. Using MRI together with synchronous MRI‐compatible EEG and nasal/oral flow measurements, this study aimed at characterizing upper airway dynamics during tidal breathing in OSA subjects and healthy controls during both wakefulness and natural sleep. We sought to test two hypotheses. First, we surmised that imaging during wakefulness may be predictive of imaging during sleep, a finding which might obviate the need for imaging during sleep in the future. We focused on tidal breathing awake and asleep rather than during respiratory events in order to test this assumption. Second, we predicted that the narrowing of the OSA airway would be more marked during sleep than that of the controls, but that the site of maximal narrowing might be variable.

## Methods

### Subjects

The study was approved by the Human Research Protection Program at the University of California at San Diego. Informed consent was obtained from each participant. Nine subjects with a clinical diagnosis of OSA and seven BMI‐matched healthy controls were recruited for this study. OSA subjects were recruited from the UCSD sleep medicine center and controls from the San Diego community. Inclusion criteria for the OSA group were moderate or severe OSA (apnea‐hypopnea index AHI > 15/h, AASM criteria) at diagnosis, no CPAP treatment within a 6‐week period preceding the study and no prior upper airway surgery. Inclusion criteria for the control group were an AHI < 5/h and no prior upper airway surgery. Absence of OSA in the control group was confirmed through Berlin and Epworth questionnaires to screen for OSA and by home sleep testing (Apnea‐Link, ResMed, Inc, California). Exclusion criteria for both groups were a body mass index (BMI) larger than 40 kg/m^2^ and contraindications to MRI (including metallic implants and claustrophobia). Sleep assessments to screen for OSA were performed within 6 weeks of the MRI study.

### Data collection

Subjects were asked to arrive at the imaging facility at a minimum of 2 h before their normal bedtime. Each subject was asked to get up earlier than his or her normal wake time the day of the study and to refrain from ingesting any caffeinated or alcoholic drinks prior to the evening imaging session. The subject was screened for MR compatibility, and filled out the Berlin and Epworth questionnaires. The subject was then fitted with ear plugs, EEG cap (Brain Products) and full facemask (Hans Rudolf, Inc.) and then positioned in the scanner in the supine position with his/her head positioned with the Frankfort plane perpendicular to the scanner table. Small cushions were inserted between the coil and the head of the subject to minimize motion during imaging.

MRI was performed on a 1.5 T General Electric (GE) HDx EXCITE clinical MRI scanner using the top C‐spine coils with the TMJ attachment. Two‐dimensional axial images were acquired, using an SSFSE (single‐shot fast spin echo) sequence with the following parameters: acquisition matrix = 128 × 256, slice thickness = 7.5 mm, TR = 2 sec, TE= 24.5 ms, flip angle = 90°, FOV= 25.6 cm, and sampling bandwith = 125 kHz. Up to 13 contiguous slices were acquired from the hard palate to the base of the epiglottis. The number of slices varied slightly between subjects because of intersubject variability in upper airway length. Every slice was acquired in pairs sequentially for up to 40 min, thus obtaining up to 1200 axial images. Subjects were imaged both during wakefulness and natural sleep.

Concurrent to MRI acquisition, quantitative flow measurements of mouth and nasal flow were recorded with MRI‐compatible pneumotachometers (Hans Rudolph Inc., Kansas City, MO) connected to a facemask along with a TTL pulse from the MRI recording the time of each image acquisition. The face mask included a separation between the nose and the mouth section (Hans Rudolph Inc., Kansas City, MO) and subjects were free to breathe through their mouth and/or nose. Blood oxygen saturation and heart rate were collected with a MRI‐compatible pulse oximeter (Nonin Medical, Inc., Minneapolis, MN, USA). Sleep was monitored with EEG by an experienced technician, using an MR‐compatible amplifier (BrainAmp MR Plus, Brain Products, Gilching, Germany) and an MR‐compatible cap (Braincap MR, EasyCap, Herrsching, Germany). The EEG, ECG and TTL pulse data was sampled at 5000 Hz with 0.016 to 250 Hz filters, and synchronized with the scanner clock, using a SyncBox (Brain Products). Electrode impedance were below 20 kΩ.

### Data analysis

The MRI gradient artifact and cardioballistic artifacts were removed from the EEG signals, using post processing software (Brain Vision Analyzer II, Brain Products). The postprocessed EEG data was then down sampled to 200 Hz and a FIR filter (cut‐off frequency = 35 Hz) applied. The final postprocessed EEG data were then synchronized and combined with the nasal and mouth flow signals, SaO_2_, heart rate by using the common TTL pulse, using MATLAB custom software. The resulting physiological files were then imported into Sleepware G3 scoring software for sleep staging.

**Figure 1:**
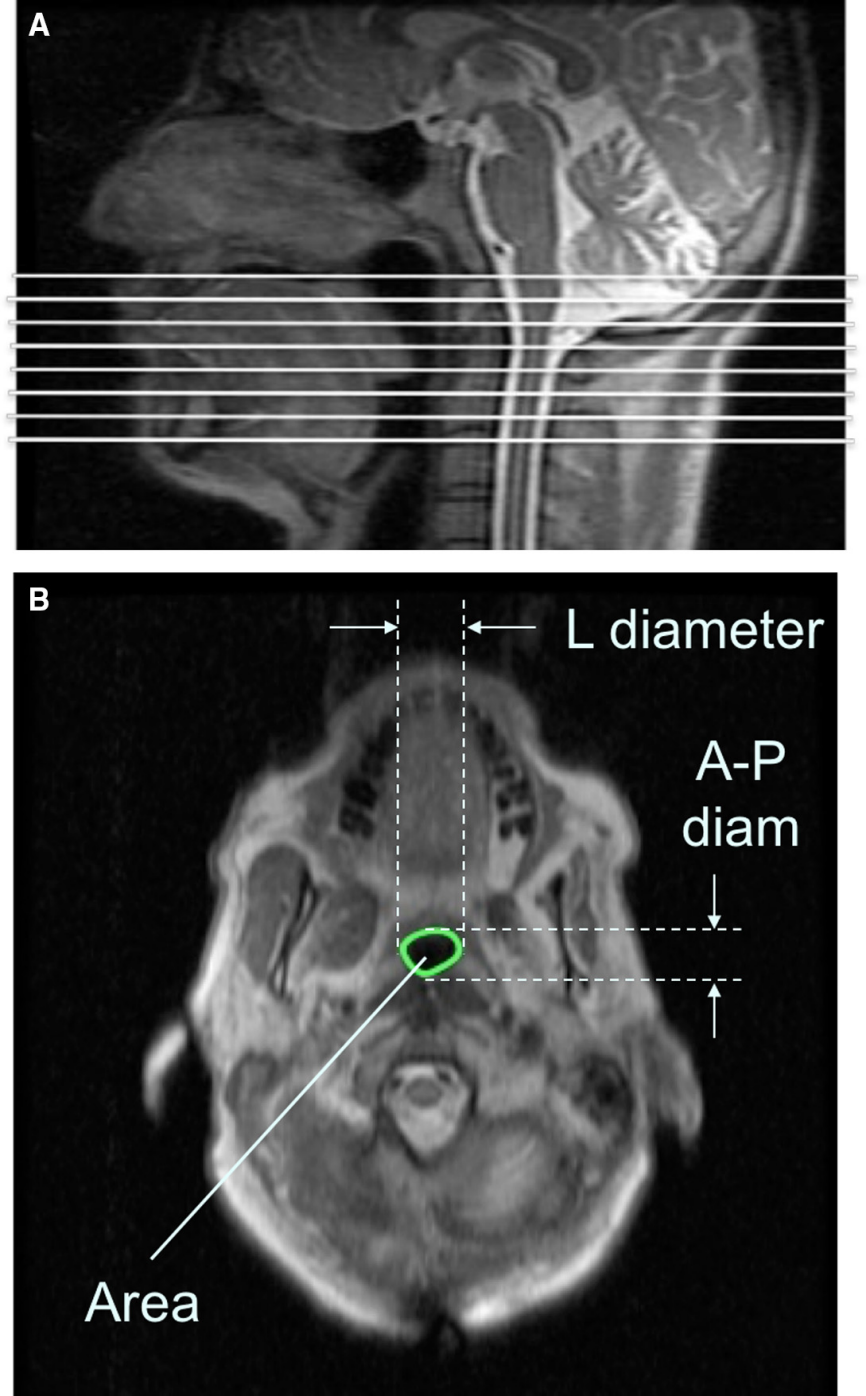
(A) Sagittal image of the upper airway showing the location of the axial slices (white lines) from the hard palate down to the epiglottis. (B) Axial magnetic resonance image of a sample subject (control), demonstrating the upper airway parameters: Area, Lateral (L) and anterior–posterior (A‐P) diameter. Diam: diameter.

The physiologic data were retrospectively scored blindly to identify sleep state and respiratory events (using Chicago criteria, Sleepware G3 scoring software), and synchronized with dynamic MR images. The image processing software Amira (Visage Imaging Inc, Carlsbad, CA) was used to identify the in‐plane area of the upper airway. The images were first sorted by slice location. The cross‐sectional area of the airway lumen was determined as all voxels having intensity below a threshold calculated on a subject‐by‐subject and slice‐by‐slice basis due to the intensity variations that are typical with multicoil acquisition (i.e., C‐spine coil). For any given slice, the subject‐based intensity threshold was calculated as the minimum intensity minus 1/2; of the SD of the tissue(s) surrounding the airway. The upper airway was then characterized by its area, and linear dimensions (lateral and anterior–posterior (A‐P)) on each image (Fig. 1), using ImageJ (U. S. National Institutes of Health, Bethesda, Maryland, USA, https://imagej.nih.gov/ij/, 1997–2016). The linear dimensions were determined by fitting the smallest possible bounding box around the airway lumen and measuring the height and width of the bounding box as in (Schwab et al. 1993b). Reliability of the analysis technique was checked by having two individuals independently analyze the same sets of images. The coefficient of correlation (r) between the two sets of upper airway dimensions was 0.96.

**Figure 2:**
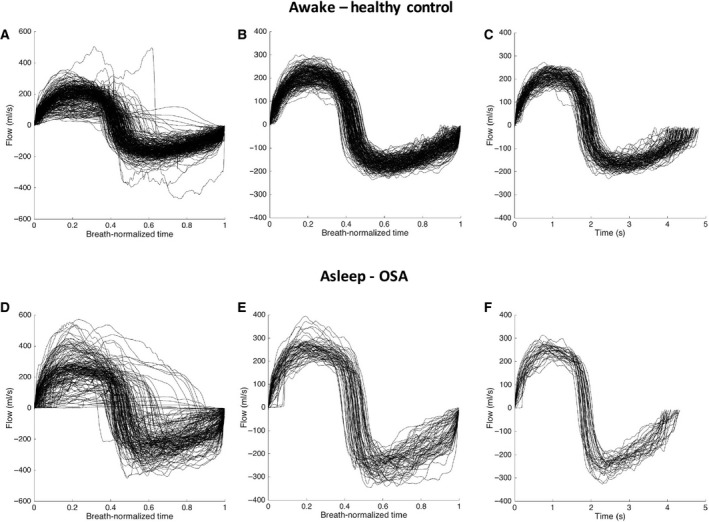
Selection of breaths used in the data analysis based on respiratory flow pattern over the images acquisition. Flow is plotted for each breath as a function of time. Panels A, B, C show data from an awake heathy control (male, 34 years old, BMI = 25.1 kg/m^2^). Panels D, E, F show data from an asleep OSA subject (male, 55 years old, BMI = 29 kg/m^2^). (A, D) flow as a function of time normalized over the duration of each breath. (B, E) data with consistent flow pattern as a function of normalized time. (C, F) flow patterns of breath within one standard deviation of tidal volume; only images acquired during these selected breaths were used in the analysis.

For each state (awake and asleep), images were sorted into an expiratory and an inspiratory group and labeled according to the level of expiration or inspiration based on flow data. Only images collected during breaths with consistent flow patterns and whose volume were within one standard deviation of the subject average tidal volume (for each state) were used in the analysis (Fig. 2). For each state (awake and asleep), data were sorted in 20% bins for both the inspiration and expiration phase and reported as average for each bin. The dynamic behavior of the upper airway was then assessed by the maximum change in airway area and linear dimensions over the tidal breath. Mean upper airway caliber was also obtained by averaging data over the tidal breath.

### Statistical analysis

Because of the small number of subjects, a nonparametrical statistical approach was used. The statistical significance of differences in anthropometric data and in each of the measured parameters at each anatomical level was determined, using Wilcoxon signed ranked tests. Significant differences were accepted at a *P* < 0.05 level. Comparison of distributions between the groups was performed, using the Kruskal–Wallis test. For any distribution that was significantly different, post hoc pairwise comparisons were performed, using the Dunn's test.

## Results

### Subjects

We studied a total of nine OSA subjects and seven BMI‐matched healthy controls. Two of the nine OSA subjects were excluded from the analyses. The first subject was excluded because of an air leak during data collection, and the second subject was excluded due to the presence of a large abnormal tissue mass in the nasal cavity. Two of the healthy controls did not fall asleep in the MR scanner. Thus, data are reported for 7 OSA subjects and 7 controls during wakefulness, and for 7 OSA subjects and 5 controls during sleep. The relevant anthropometric data of the subjects included in the analysis are listed in Table 1.

**Table 1:** Demographic data and sleep variables – Median (minimum, maximum)

	Controls (*N* = 7)	Apneic (*N* = 7)	*P*‐value
Gender	6M/1F	6M/1F	
Age (yr)	45 (28, 63)	58 (41, 66)	0.03
BMI (kg/m^2^)	25.0 (21.9, 34.4)	29.0 (24.1, 31.6)	0.24
Neck circumference (cm)	36.7 (34, 38.7)	39.6 (32, 41.9)	0.12
AHI (events/h)	3.0 (1.5, 5.6)	60.8 (35.0, 79.6)	<0.0001

BMI, body mass index, AHI, apnea‐hypopnea index.

There were no significant differences in BMI and neck circumference between the OSA and the control group (Table 1). Both AHI and age were significantly higher in the OSA subjects than in the control group. There were no demographics differences between OSA and controls after excluding the two controls that did not sleep in the scanner from analyses examining sleep‐related metrics.

There were no significant differences in tidal volumes between groups both during wakefulness (controls (*N* = 7): 635 ± 162 mL, OSA (*N* = 7): 592 ± 130 mL, *P *= 0.60) and sleep (controls (*N* = 5): 544 ± 157 mL, OSA (*N* = 7): 588 ± 138 mL, *P* = 0.59). Most of the subjects were nose breathers with the exception of one subject in each group that were breathing simultaneously through their mouth and nose, and one healthy control who was a mouth breather. In all subjects (both groups), only sleep stages (N1 or N2) were recorded. No subject showed deep sleep stages (N3 or N4) or REM sleep. On average, subjects slept for 20 min in the scanner with 75% of images recorded during N1 stage.

### Change in upper airway size during tidal breathing

**Figure 3:**
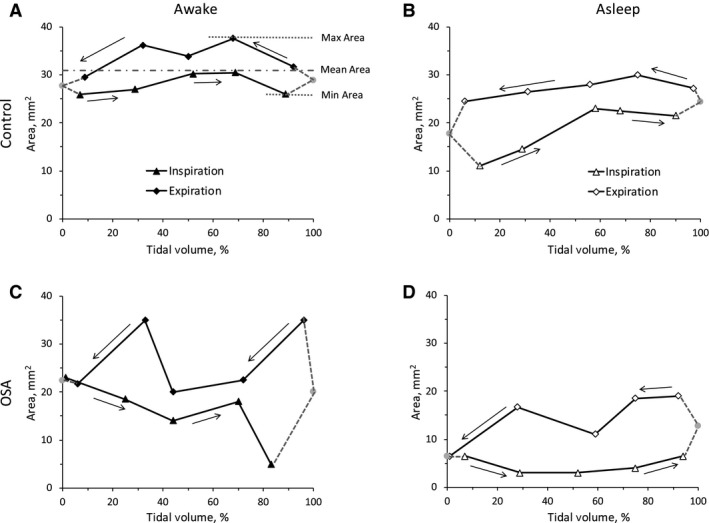
Variation in upper airway cross‐sectional area at the low retropalatal level as a function of tidal volume. Data are shown in one control (awake: panel A, asleep: panel B) and one OSA subject (awake: panel C, asleep: panel D). Solid line with triangles = inspiration, solid line with diamonds = expiration. Dotted lines with solid circle = extrapolation between inspiration and expiration measured data.

Data obtained during N1 and N2 stages were pooled together in the analysis to ensure a sufficient number of data points to describe variations in upper airway dimensions during asleep tidal breathing in each slice. There were significant dimensional changes in upper airway parameters during tidal breathing with larger values seen during expiration than during inspiration. As an example, variation in upper airway size at the low retropalatal level is shown in Figure 3 for a representative OSA subject and a representative control during both wakefulness and sleep (N1 stage).

**Figure 4:**
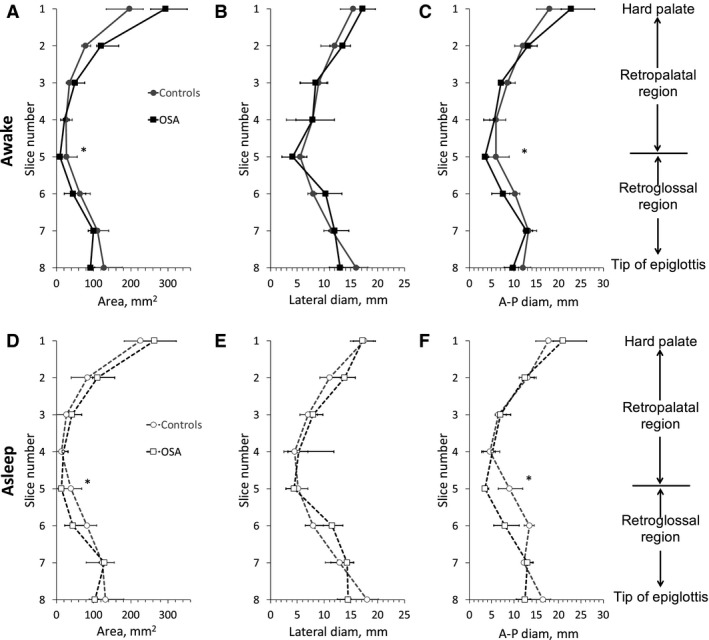
Minimum dimensions of the upper airway during tidal breathing in awake (panels A–C) and asleep (panels D–F) OSA subjects (square symbols) and controls (circle symbols). (A, D) Area. (B, E) Lateral diameter. (C, F) anterior–posterior (A‐P) diameter. Data are shown for nose‐breathers only (Controls: *N* = 5 (awake), *N* = 4 (asleep); OSA: *N* = 6 (awake and asleep)) and described by the median and interquartile range (25th–75th percentile). *Significantly different between groups (*P* < 0.05).

Minimum dimensions of the upper airway during tidal breathing are shown in Figure 4 for in each group (nose breathers only) both while awake (Fig. 3A–C, OSA: *N* = 6, controls, *N* = 5) and during sleep (Fig. 3D–F, *N* = 4 for controls and *N* = 6 for OSA subjects). During both wakefulness and sleep, the area and the anterior–posterior (A‐P) diameter of the upper airway was smaller in the OSA group than in controls both in the low‐retropalatal/high‐retroglossal region (slice 5, Fig. 4A, C, D and F). There was no difference in the lateral diameter of the upper airway between groups in both states (Fig. 4B and E).

**Figure 5:**
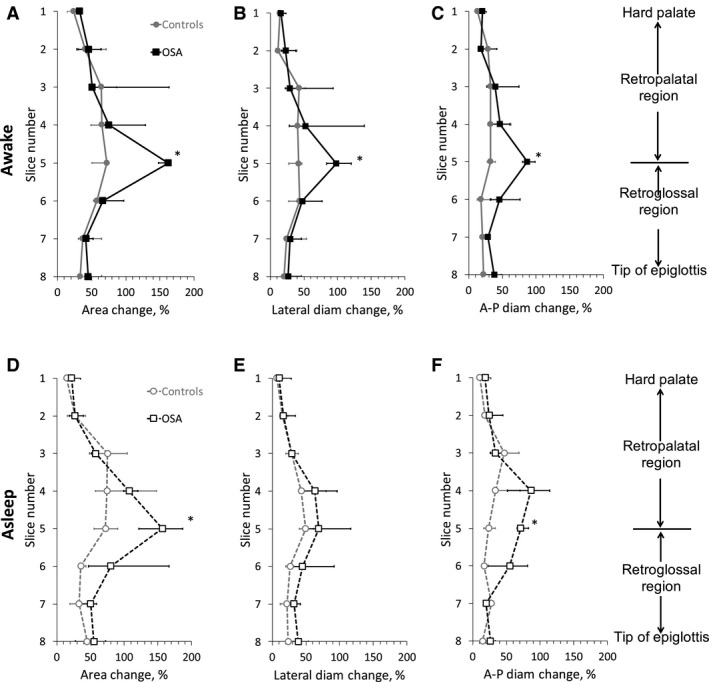
Normalized change in the upper airway dimensions over a tidal breath in OSA subjects (square symbols) and controls (circle symbols). Data are shown for nose‐breathers only (Controls: *N* = 5 (awake), *N* = 4 (asleep); OSA: *N* = 6 (awake and asleep)) and described by the median and interquartile range (25th–75th percentile) in each group. (A, D) Area. (B, E) Lateral diameter. (C, F) anterior–posterior (A‐P) diameter. *Significantly different from control (*P* < 0.05). See text for details.

The dynamic behavior of the upper airway during tidal breathing is also shown in Figure 5 both during wakefulness and sleep. Dynamic changes were represented as the maximum change in upper airway dimensions (area and linear dimensions) over the tidal breath normalized by the mean dimension (i.e., (max − min)/mean, see Fig. 3A). The largest changes between minimum and maximum area were found in the low retropalatal/ high retroglossal region and these changes were significantly larger in the OSA than in the control group (*P* < 0.05). These changes were observed both while awake and during sleep.

### Upper airway dynamics during wakefulness as a predictor of airway collapse during sleep

**Figure 6:**
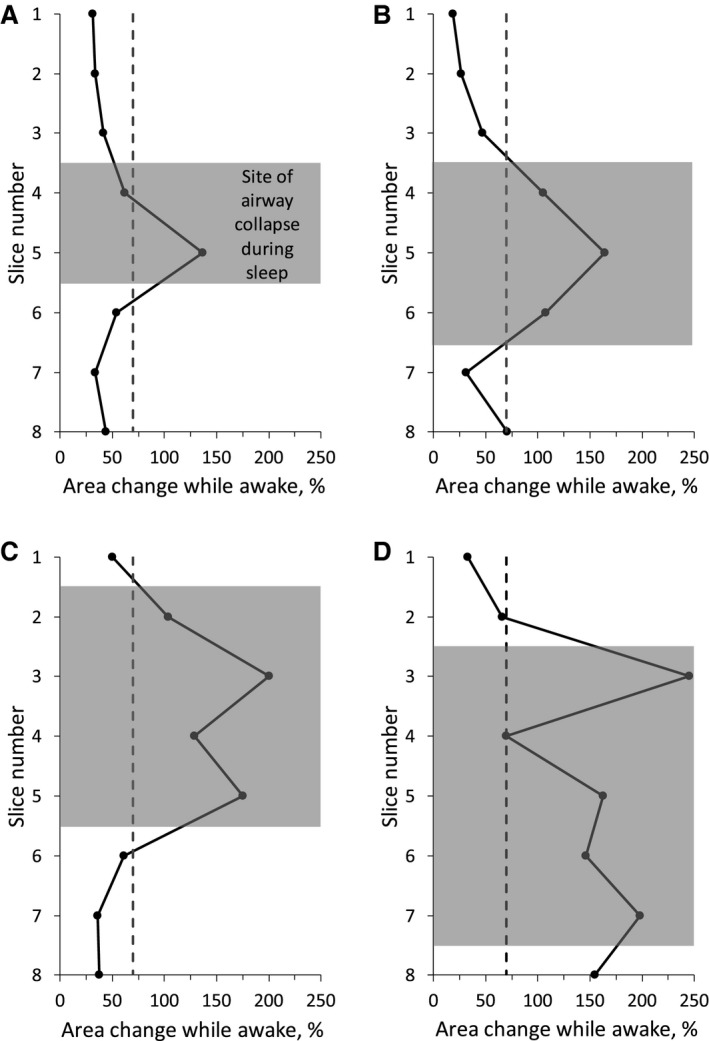
Upper airway dynamics during awake tidal breathing in four OSA subjects. Gray area identifies the site of upper airway collapse during obstructive apneas experienced by the subjects. See text for details.

Four of the OSA subjects we studied experienced obstructive apneas during MR imaging. There was variability among these subjects in the site and length over which the airway collapsed during an obstructive apnea event with the obstruction extending over 2 (~ 15 mm, Fig. 6A), 3 (~22 mm, Fig. 6B), 4 (~30 mm, Fig. 6C) or 5 slices (~ 37 mm, Fig. 6D). In three of the subjects, the airway collapsed in the retropalatal region while in the fourth subject the collapse extended over both the retropalatal and retroglossal regions. In all cases, the site of collapse occurred in slices that exhibited the largest dynamic change during awake tidal breathing (see Fig. 6 where the shaded area indicates the site of upper airway collapse during obstructive apneas).

## Discussion

Using MRI together with synchronous MRI‐compatible EEG and nasal/oral flow measurements, we characterized upper airway dynamics during tidal breathing in OSA subjects and BMI‐matched healthy controls during both wakefulness and natural sleep. This study offers a number of findings. First, there was no major difference in the upper airway structure between OSA and controls except for a narrower airway at the low‐retropalatal/high‐retroglossal level in OSA than in controls during both wakefulness and sleep (Fig. 4). Anterior–posterior rather than lateral narrowing was seen in OSA patients when compared to matched controls. Second, changes in upper airway size during tidal breathing were more marked in OSA patients compared to matched controls (Fig. 5). Third, the site of upper airway collapse in OSA subjects during sleep was associated with the region of the upper airway where changes in caliber during awake tidal breathing were the greatest (Fig. 6).

Several authors have previously described upper airway structure mainly during wakefulness. Schwab et al. (2003) reported significant difference in upper airway volume and cross‐sectional area between awake controls and awake OSA subjects, which contrast with the findings from this study. The discrepancy is likely because, unlike subjects from this study (Table 1), controls and OSA subjects were not matched in terms of BMI in Schwab's study with OSA subjects having a significantly higher BMI than controls (36.2 ± 8.8 vs. 25.9 ± 4.8, *P* < 0.001 (Schwab et al. 2003)). One should also note that age may be a contributing factor to the difference between the two studies, as OSA subjects were older than the controls in the present study unlike the subjects from Schwab's study. Upper airway caliber has been shown to decrease with increasing age, however, the effect is relatively small. Indeed, in a group of 114 healthy normal adults, Martin et al. (1997) showed that age explains no more than 13 % of the variance seen in upper airway caliber. Yet, as in the present study, Schwab's group also showed a significant change in dynamic behavior at the retropalatal and retroglossal level between awake OSA subjects and normal controls (Schwab et al. 1993a).

Recent literature has emphasized the multifactorial nature of OSA pathogenesis. A number of factors underlie the occurrence of OSA in various individuals suggesting a potential role for personalized medicine in these patients. For example, upper airway surgery is unlikely to be beneficial for OSA patients in whom the mechanism of apnea is related to unstable ventilatory control. Similarly, hypoglossal nerve stimulation to increase upper airway muscle tone may not be helpful to patients with OSA due to severe anatomical compromise. On the other hand, hypoglossal nerve stimulation to increase genioglossus muscle activity may well be helpful for select patients with narrow AP diameter particularly if observed at the level of the retroglossal airway. At present, no definitive method is available to predict a priori the response to a given intervention, emphasizing the need for further research.

The shape of the upper airway has been discussed in the literature by Collard et al. (1996), Leiter (1996) and Schwab et al. (2003). The exact role of pharyngeal shape in OSA pathogenesis remains unclear. Early literature by Schwab et al. (1995) emphasized the importance of the lateral pharyngeal walls in mediating pharyngeal collapse. However, these authors have more recently emphasized the importance of tongue fat which one could argue is more likely to mediate AP rather than lateral collapse of the upper airway. As such, we are supportive of further efforts to define various mechanisms of upper airway collapse, since we predict that these differences may mediate different patterns of airflow limitation and could be amenable to individualized therapeutic interventions.

The level of anatomical obstruction may also have important implications. Prior authors have suggested no major correlation between the site of airway narrowing in wakefulness as compared to sleep. In theory, narrowing at the level of the velopharynx may be amenable to uvulopalatopharyngoplasty whereas AP narrowing at the retroglossal airway may respond well to hypoglossal nerve stimulation. Our new findings suggest a group of OSA patients with retroglossal AP narrowing who may respond well to efforts to augment pharyngeal dilator muscle tone. Our observation of increased tidal changes in airway structure in OSA versus matched controls is also of note. This finding may be indicative of increased upper airway compliance although we recognize that differences in surface forces and dilator muscle activity may also have a role. Therefore, we prefer the term “effective elastance” to summarize the observed differences in OSA versus matched controls. Also, the observation that dynamic imaging during wakefulness differs in OSA versus controls may suggest a role for this approach in the clinical assessment of pharyngeal properties. Indeed, the site of airway collapse during sleep in the OSA group was associated with the region of the upper airway where changes in caliber during awake tidal breathing were the greatest (Fig. 6).

Despite our novel findings, we recognize a number of limitations. First, our sample size was quite modest and this may affect the lack of statistical significance in some of the reported parameters. Also, the effect of possible confounding factors such as comorbidities could not be reliably assessed in this small sample size. We emphasize the labor‐intensive nature of gathering MR images during natural sleep while assessing respiratory pattern; thus, we agree with the need for larger studies, but view our new findings as a first step that established this complex approach as a beneficial method to gain greater insight in upper airway dynamics. Second, we did not make rigorous mechanical measurements such as the critical opening pressure (P_crit_) in our participants as we typically perform in our physiology laboratory. Such experiments are challenging to perform in an MR scanner and thus we agree that further study is required. Moreover, we acknowledge that some of our assertions in our discussion above are speculative. Third, we have not made therapeutic interventions based on our imaging observations and thus our ability to predict such responses is also speculative. Nonetheless, we assert that interventional studies have both risk and expense which cannot be justified until studies such as the present one have been performed. Fourth, one could argue that by focusing on tidal breathing, we have excluded apneas and severe hypopneas which occur in OSA but not in controls. This process may have biased our results by excluding the narrowest airways from the OSA group. As such, we believe that this approach may have biased towards the null hypothesis (making OSA look more similar to controls) making any observed differences between groups more robust. However, data from a subset of OSA subjects who experienced obstructive apneas during imaging provide strong support that upper airway dynamics during awake tidal breathing can inform on site of upper airway collapse during sleep. Thus, despite the limitations of this study, we think our new findings provide a good basis for further investigation in a larger number of subjects to confirm the generalization of the findings.

## Conflict of Interest

ResMed, Inc. provided a philanthropic donation to the University of California, San Diego (UCSD) in support of the UCSD sleep center. No other conflicts of interest, financial or otherwise, are declared by the author(s).
